# Long-Term Effects of Periodical Fires on Archaeal Communities from Brazilian Cerrado Soils

**DOI:** 10.1155/2019/6957210

**Published:** 2019-01-27

**Authors:** Aline Belmok, Thiago Rodrigues-Oliveira, Fabyano A. C. Lopes, Heloisa S. Miranda, Ricardo H. Krüger, Cynthia M. Kyaw

**Affiliations:** ^1^Institute of Biological Sciences, Department of Cell Biology, University of Brasilia, 70910-900 Brasília, DF, Brazil; ^2^Institute of Biological Sciences, Department of Biochemistry and Molecular Biology, Federal University of Goiás, 74690-900 Goiânia, GO, Brazil; ^3^Institute of Biological Sciences, Department of Ecology, University of Brasilia, 70910-900 Brasília, DF, Brazil

## Abstract

The Cerrado biome corresponds to an extensive area of Brazil and is considered a biodiversity hotspot. Frequent fires are a natural feature in this biome and have influences on vegetation structure and composition. However, continuous anthropogenic actions are promoting changes in fire frequency and seasonality. Despite the high biodiversity of the Cerrado, little is known about its microbiome, with few publications describing some aspects of the bacterial and fungal communities found on this biome and almost no references about archaea. In this study, we describe the archaeal diversity in Cerrado *sensu stricto* soils, comparing the archaeal communities from soils of an area long protected from fires to one exposed to biennial fires, using both 16S rRNA and *amoA* genes as molecular markers. Almost all 16S rRNA sequences from both studied areas were affiliated with I.1b and 1.1c *Thaumarchaeota*, groups commonly detected in terrestrial environments. A higher relative abundance of I.1b thaumarchaeal subgroup was detected in the frequently burned area even though no statistically significant differences were observed in archaeal 16S rRNA richness and diversity between the investigated areas. Many ammonia-oxidizing archaea (AOA) are affiliated with this group, which is consistent with the higher *amoA* diversity and OTU numbers detected in the area periodically burned. Taken together, our results suggest that, although total archaeal community richness and diversity do not seem to greatly differ between the investigated conditions, alterations in wood cover and vegetation structure caused by frequent fires likely cause long-term effects in AOA diversity in Cerrado soils.

## 1. Introduction

It is well established that organisms of the *Archaea* domain are ubiquitously distributed and represent a significant fraction of the prokaryotic cells found in aquatic and terrestrial ecosystems [1, 2]. In general, archaea can account for 1-2% of total prokaryotes in moderate aerobic soils [3], reaching up to 5% in sandy soils [4] and even 38% in acidic forest soils [5]. Furthermore, the roles of soil archaea in the nitrogen cycle have been increasingly debated [6–8], with the frequent detection of ammonia-oxidizing archaea (AOA) in these environments [9, 10]. Although most archaea found in soils (and other mesophilic environments) were initially classified as Group I Crenarchaeota [11], they are now classified as members of the Thaumarchaeota phylum [12], which contains all AOA known so far [13].

Brazil is a country widely known for its continental proportions, with many different terrestrial landscapes and biomes. However, in spite of its notable biodiversity, studies focusing on archaeal diversity in natural Brazilian environments are still scarce, with only about 50 studies published in the last 20 years according to a recent review by Rodrigues et al. [14]. Most of these studies investigated archaeal communities in soils from the Amazon or Atlantic Forest biomes, but very few have focused on archaeal diversity of the Brazilian savanna, also known as Cerrado [15–19]. This biome is extensive and extremely diverse, corresponding originally to approximately 22% of the country's territory. Due to the large number of endemic species and the continuous degradation of its habitats, the Cerrado is considered a hotspot for conservation priorities [20]. This biome presents different physiognomic forms, ranging from grassland to forest-like vegetation [21]. The most common physiognomy is the Cerrado *sensu stricto*, a savanna woodland with a continuous herbaceous layer of scattered trees and shrubs and canopy closure ranging from 20 to 50% [21]. The seasonality of rains, poor nutrient soils, and fires are considered the determinants of this biome vegetation [22, 23].

Natural fires occurred in the Cerrado for millennia [24] and are still present mostly during the transition from dry to wet season [25, 26]. However, continuous occupation and conversion of large areas of Cerrado to agriculture and pasture have increased fire frequency and altered the fire season. Although many Cerrado species have adaptations to fire [22], changes in fire regimes strongly influence the structure and composition of Cerrado's vegetation, mostly due to mortality [27, 28], topkill and resprouting from underground organs [29, 30], alteration in population dynamics resulting from the loss of reproduction investment and seedling mortality [31, 32], and significant increase in grass cover [33]. The large reduction in canopy cover results in alterations in nutrient cycling mostly due to the reduction of litter formation [34], changes in temperature due to gaps in the herbaceous layer vegetation [35], and consequently, changes in soil humidity [36, 37], all of which may alter the composition of archaeal communities [38–40].

In this work, we present a case study to investigate if the archaeal communities from typical Cerrado *sensu stricto* areas with long protection from fires differ from those of areas submitted to biennial fires in mid dry season. Soil samples were collected from areas of well-known fire history. Twelve DNA libraries were obtained and sequenced, six using the 16S rRNA *Archaea*-specific gene and six using the archaeal *amoA* gene. The resulting sequences were analyzed to evaluate the differences between archaeal communities in Cerrado *sensu stricto* soils from frequently burned and fire-protected areas.

## 2. Material and Methods

### 2.1. Study Site and Soil Sampling

The study areas are located in the Ecological Reserve of the Brazilian Institute of Geography and Statistics (RECOR-IBGE), 35 km south of Brasilia, DF (15°55′S, 47°51′W). The Reserve has an area of 1360 ha, encompassing the most common Cerrado physiognomies. The climate in the area is Cwa in Köppen's classification with two well-defined seasons: a dry season lasting from June to October and a rainy season when most of the average precipitation of 1436 mm occurs. Mean temperatures are around 21°C, and the altitude varies from 1048 to 1150 m. The soil is typically yellow-red latosol (Acrustox according to the American classification) [41].

**Figure 1:**
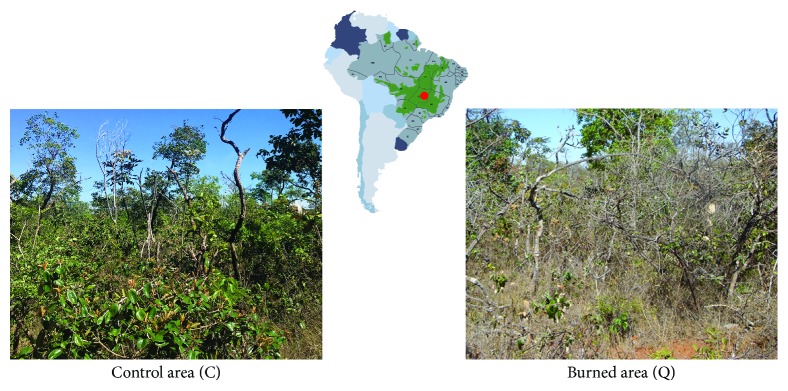
Location of Cerrado *sensu stricto* sampling sites (indicated in red), at the Reserva Ecológica do IBGE, Brasilia, Brazil. The distribution of the Cerrado biome is indicated in green on the map. The Control area (C, on the left), that has been protected from fires for 20 years, and the Burned area, submitted to a biennial fire regime since 1992 (Q, on the right), are highlighted.

In September 2013, two Cerrado *sensu stricto* areas with contrasting and well-known fire history were selected for this work. The first area was protected from fire for 20 years (Control area = C), and the second, 4 m apart, was submitted to a biennial fire regime in the mid dry season (August) from 1992 to 2008 (Burned area = Q, from the Portuguese word for burned—*Queimada*) (Figure 1). In September 2011, a wildfire burned Q without affecting C. Therefore, Q was protected from fire for 2 years prior to our study. Canopy cover in C and Q varied from 32% to 84% and from 28% to 64%. The aboveground biomass of the herbaceous layer in C was 6.3 ± 1.4 Mg/m^2^, with the grasses and forbs representing 3% and 12% of the total. The dry mass of litter was 5.4 Mg/ha. Total herbaceous biomass in Q was 7.7 ± 1.1 Mg/m^2^, with the grasses and forbs representing 35% and 26% of total, and the dry mass of litter was 3.0 Mg/ha.

In each area, three soil samples were randomly collected, within a 10 m × 10 m plot, being at least 1 m apart from each other. The soil was collected to a depth of 10 cm with a PVC tube, 10 cm in diameter, and stored at −20°C until used. Physicochemical properties for the soil samples of each area were performed by SoloQuímica Análises de Solo Ltda. Principal component analyses (PCA) were performed on abundance measures using a correlation matrix in Factoextra v.1.0.3 package implemented by R v.3.2.2 [42]. Statistical differences in soil physical-chemical properties were compared by Welch's *t*-test (*α* < 0.05) using R statistical software v.3.2.2 [42].

### 2.2. DNA Extraction and 16S rRNA and *amoA* Gene Library Construction

Total soil DNA was extracted from 0.5 g of each sample with PowerSoil DNA Isolation Kit (MO Bio Laboratories Inc.) according to the manufacturer's instructions. PCR assays were conducted with primer pairs 21f/958r [43] and Arch amoAf/Arch amoAr [44], for archaeal 16S rRNA and *amoA* genes, respectively. All PCR assays were performed in 50 *μ*L reaction mixtures, containing 1 to 100 ng of template DNA, 1X reaction buffer (Invitrogen^®^), 1.5 mM MgCl_2_ (Invitrogen^®^), 400 ng/*μ*L bovine serum albumin, 0.5 *μ*M of each primer, 200 *μ*M dNTPs (Invitrogen^®^), and 1.25 U *Taq* DNA Polymerase (Invitrogen^®^). The PCR experiments were performed in a MJ PTC-100^®^ (Peltier Thermal Cycles) thermocycler, with cycling conditions previously described [43, 44]. Amplified DNA was visualized on 1% agarose gel electrophoresis stained with ethidium bromide (10 mg/mL). Amplicons were purified using GeneJET PCR Purification kit (Thermo Scientific^®^), cloned into the pGEM-T Easy^®^ (Promega) vector, according to manufacturer's instructions, and transformed into *Escherichia coli* DH5*α* competent cells by heat shock treatment. Plasmidial DNA of the recombinant clones was extracted by phenol-chloroform-isoamyl alcohol at 25 : 24 : 1 (vol/vol/vol) and was sequenced by the Sanger method at Macrogen Inc. (Korea).

### 2.3. Phylogenetic and Statistical Analyses

All sequences were trimmed according to Phred quality superior to 20 in more than 400 nucleotides. 16S rRNA gene sequences were used for comparative analyses with the Greengenes taxonomical database [45] using Mothur v.1.24.1 [46] with an identity threshold of 90% or higher. Although Greengenes does not consider Thaumarchaeota as a separate phylum (it is considered as a crenarchaeotal class), in this work we considered it in the phylum taxonomical level as suggested by Brochier-Armanet et al. [12]. Multiple alignments of 16S rRNA and *amoA* gene sequences were performed with Clustal X v. 2.1 [47]. Gap columns generated by the alignment processes were filtered using Mothur v.1.24., also employed for rarefaction curve estimations. For calculations of coverage, richness, and diversity indexes—Ace, Chao, and Shannon [48–50]—libraries were normalized for the number of sequences of the smallest dataset for each gene (*n* = 50 for 16S rRNA and *n* = 30 for *amoA*) and significant differences in indicators were evaluated by Welch's *t*-test (*p* < 0.05). Analysis of both 16S rRNA and *amoA* gene sequences was OTU-based and the identity threshold adopted was 97% nucleotide sequence similarity [13, 51].

Relative abundances of 16S rRNA OTUs from each area were evaluated using STAMP v.2.1.3 [52]. The areas were compared by Welch's *t*-test (*p* < 0.05), and nonmetric multidimensional scaling (NMDS) for each profile was performed using the Bray-Curtis similarity index in R software [42]. To construct phylogenetic trees with both 16S rRNA and *amoA* genes, multiple alignments of the deduced sequences were performed in MUSCLE v. 3.8.31 [53] using default parameters and manually edited. The phylogenetic trees were constructed in FastTree v.2.1 with default parameters and 1,000 bootstrap test [54].

## 3. Results

### 3.1. Soil Physicochemical Characteristics

**Figure 2:**
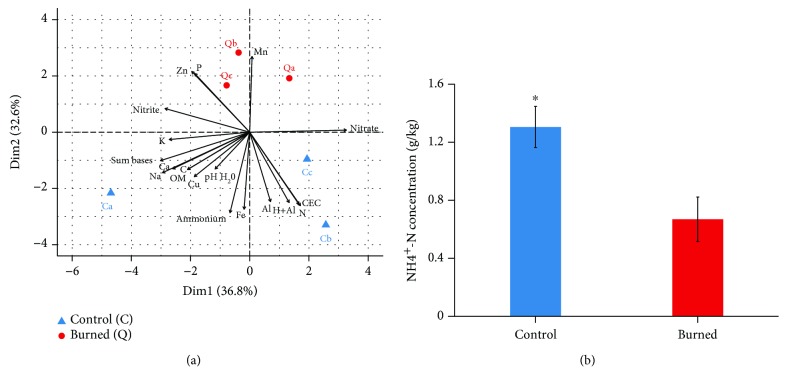
Principal component analysis (PCA) of soil physicochemical properties based on a correlation matrix performed in Factoextra R package v.1.0.3 (a). OM: organic matter; CEC: cation exchange capacity. (b) NH_4_^+^-N concentrations in soils from Control and Burned areas, with asterisk indicating significant difference detected in Welch's *t*-test.

The physicochemical properties of Cerrado *sensu stricto* soils (Supplementary Table 1) revealed an acidic pH (around 4.9) in both Control and Burned areas. Principal component analysis (PCA) of soil physicochemical data (Figure 2(a)) indicated separation of soil samples from Control and Burned areas in the first dimension axis, suggesting higher influence of Mn in soils from the Burned area and Fe and ammonium in samples from the Control area. However, Welch's *t*-test revealed that, among all physicochemical parameters analyzed, only NH_4_^+^-N was significantly different in the studied sites, with higher concentrations in the Control area (Figure 2(b)).

### 3.2. Archaeal 16S rRNA Gene Analysis

DNA amplifications of the archaeal 16S rRNA gene resulted in six clone libraries: three replicates from the Control area (Ca, Cb, and Cc) and three from the biennially Burned area (Qa, Qb, and Qc). In total, 466 sequences with high Phred quality (>20) over 400 bp were obtained from these six libraries (Supplementary Table 2), all classified as *Archaea* according to the Greengenes taxonomical database, resulting in 32 OTUs with a 97% sequence similarity cutoff.

**Figure 3:**
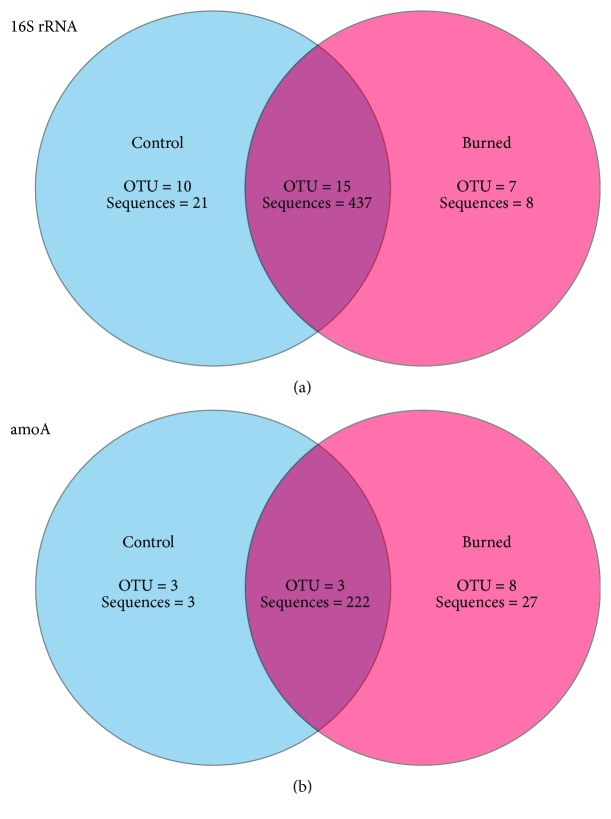
Venn diagrams showing unique and shared archaeal 16S rRNA (a) and *amoA* (b) OTUs (97% sequence similarity) between Cerrado *sensu stricto* soils from the area protected from fire for 20 years (Control) and the area submitted to biennial fire regime (Burned).

Rarefaction analysis (Supplementary Figure 1) and high coverage estimators (Table 1) indicate that archaeal communities in typical Cerrado *sensu stricto* soils from both study sites were well covered, even at OTUs with 97% sequence similarity. A comparable number of OTUs were observed in each area with no statistically significant differences detected in Ace, Chao1, and Shannon values, suggesting similar archaeal richness and diversity in soils from both areas (Table 1). Furthermore, the majority of OTUs (15) identified in soils from the Control area (representing most of the sequences obtained) were also observed in soils from the Burned area, reinforcing the similarity of the archaeal community inferred by 16S rRNA genes in soils from both conditions analyzed (Figure 3(a)). These results are in accordance with those obtained in nonmetric multidimensional scaling (NMDS) analysis performed with this marker gene, where no clear clustering pattern among replicates from the Control and Burned soils could be detected (Supplementary Figure 2).

**Figure 4:**
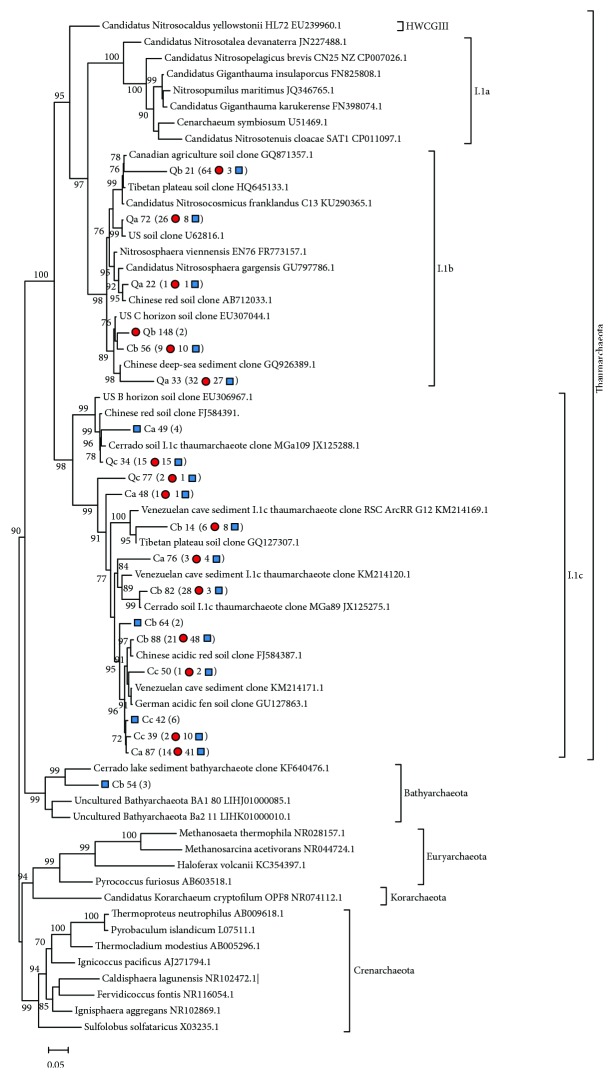
Phylogenetic tree of archaeal 16S rRNA gene OTUs (97% sequence similarity) obtained from typical Cerrado *sensu stricto* soils. The number of sequences represented by each OTU is shown in parentheses, with those from the Control area (C) indicated by blue squares and from the Burned area (Q) by red circles. Singleton sequences were not included. The tree was constructed using FastTree v. 2.1 [54], with 1000 bootstrap tests. Values below 70% are not shown. HWCG III: hot water crenarchaeotic group III.

Almost all 16S rRNA gene sequences (99.36%) were affiliated to either I.1b or I.1c subgroups of the phylum Thaumarchaeota and only three sequences (0.64%), all from the replicate Cb, were identified as belonging to the Bathyarchaeota phylum (Figure 4). It is noteworthy that, despite the similarity in richness indexes (Table 1) and numbers of observed OTUs shared between both studied conditions (Figure 3(a)), a higher relative abundance of I.1c thaumarchaeal subgroup sequences (77.68%) was retrieved from soils sampled in the Control area. In contrast, most of the sequences detected in soils from the frequently burned area were affiliated with I.1b Thaumarchaeota (58.80%), as evidenced in Figure 4. Furthermore, with the exception of Cb 54, all OTUs composed exclusively by sequences obtained from Control soils clustered within the I.1c thaumarchaeal clade (Figure 4).

### 3.3. Archaeal *amoA* Gene Analysis

Archaeal *amoA* gene libraries were obtained from the same soil samples, resulting in 251 sequences with high Phred quality (>20) over 400 bp (Supplementary Table 2). A total of 12 OTUs were identified, using a 97% sequence similarity cutoff. Coverage estimators indicated that sampling efforts were also satisfactory when using this gene (Table 2). Unlike the results obtained for 16S rRNA sequences, significant differences were detected in Shannon index values for this marker gene (Table 2), suggesting higher *amoA* diversity in soils from the biennially burned area. In addition, despite the fact that most of the archaeal *amoA* sequences grouped in three highly representative OTUs could be detected in both Control and Burned soils (Figure 3(b)), a number of OTUs were observed only in soils from the Burned area, indicating a possible occurrence of unique AOA in soils submitted to a biennial fire regime.

**Figure 5:**
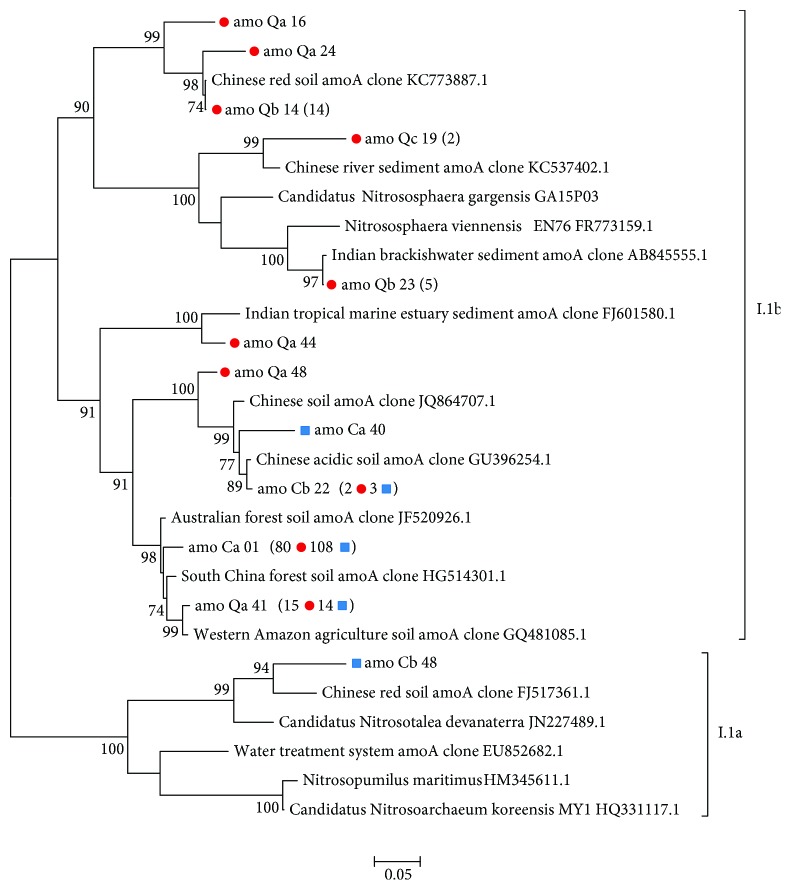
Phylogenetic tree of archaeal *amoA* gene OTUs (97% gene sequence similarity) obtained from typical Cerrado *sensu stricto* soils. The number of sequences represented by each OTU is shown in parentheses, with those from the Control area (C) indicated by blue squares and from the Burned area (Q) by red circles. The tree was constructed using FastTree v. 2.1 [54], with 1000 bootstrap tests. Values below 70% are not shown.

The phylogenetic tree constructed with *amoA* OTUs revealed that most sequences clustered with AOA from the I.1b subgroup, with the exception of Cb 48 (Figure 5). Curiously, although no 16S rRNA gene sequences were classified as I.1a thaumarchaeota, this *amoA* OTU was affiliated to this subgroup. Excluding Qb 23 and Qc 19, two OTUs representing only sequences from the Burned area that were closely related to *Nitrososphaera* spp., all other OTUs clustered in clades composed exclusively by uncultured AOA. It is worth highlighting that among these OTUs, Ca 01 contains 85.03% of Control area sequences, being extremely representative of this area's AOA diversity.

## 4. Discussion

In this study, we evaluated the long-term effects of frequent fires on soil archaeal communities from the Brazilian Cerrado. Fire is a determinant in this biome, with records of significant wildfires and fire utilization by native populations dating back thousands of years [55]. However, increasing human occupation and agricultural activities in the past decades have led to changes in fire frequency and seasonality, and although many alterations in Cerrado's vegetation structure and composition have been reported [27, 33, 56], information about the long-term effects of periodical fires in microbial communities from soils are still scarce.

The soils investigated in this study were sampled in areas of well-known fire history, where a large experiment on the effects of prescribed burnings has been conducted since 1990. With the exception of ammonium concentrations, no significant differences were detected in most physicochemical properties of soils from the Control and Burned areas (Supplementary Table 1), which is in accordance with studies previously conducted in similar sites of this ecological reserve [33]. Although short-term alterations in soil nutrient availability after fires have been described [22, 57], Cerrado soils are very resilient [22]. Since the aim of this work was to investigate long-term effects of frequent fires, soils from the Burned area were sampled two years after the last burning event, which could explain the similarity observed in most soil properties from both analyzed areas.

Almost all 16S rRNA gene sequences obtained in this study were affiliated to I.1b and I.1c subgroups of Thaumarchaeota. I.1b thaumarchaeotes are dominant in numerous terrestrial habitats [1, 39], and the ability to oxidize ammonia has been identified in many of its members, suggesting an important role of this group in nitrogen cycling in these environments [8, 10, 58]. The I.1c thaumarchaeal lineage was originally described in boreal Finnish forest soils [59], and later 16S rRNA sequences associated to this group have been detected in many other acidic soils [60–62], a characteristic feature of Cerrado [63]. So far, there are no cultured representatives of this group, and although in situ microcosm experiments indicated that these thaumarchaeotes are not associated to ammonia oxidation [64], their ecological roles are still obscure. It is worth mentioning that sequences affiliated to I.1b and I.1c groups have been detected in soils from other Cerrado's vegetation physiognomies, indicating that these organisms could be common in these soils [15].

A single OTU detected in the Control area, representing a small fraction of 16S rRNA sequences (0.64%), was closely affiliated to a bathyarchaeote clone retrieved from Cerrado lake sediments [65]. Bathyarchaeota (previously known as Miscellaneous Crenarchaeotic Group: MCG) sequences are abundant in anoxic terrestrial and aquatic environments [66, 67], and despite the current lack of cultured representatives, metagenomic data have suggested wide metabolic capabilities in members of this phylum [68], including possible involvement in aromatic compound degradation [69], protein remineralization [70], and methane metabolism [71]. Although bathyarchaeote 16S rRNA gene sequences have been identified in other Brazilian biome soils, such as the Atlantic Forest peatlands [16] and native Amazonian forest [19], members of this phylum have not been previously detected in Cerrado soils. This fact allied to the small number of sequences of this group detected in the present study may suggest a low abundance of Bathyarchaeota in these soils. However, it is important to highlight that sampling depth and period (dry or rainy seasons) may influence these results, since members of this phylum seem to predominate in deeper or highly humid soils [66].

It is worth mentioning that, despite the similarity observed in archaeal richness and diversity inferred by 16S rRNA gene analyses (Table 1), the distribution of thaumarchaeal sequences in Control and Burned areas suggested a higher relative abundance of the I.1c group in the site long protected from fire. A predominance of I.1c thaumarcheota has been commonly reported in more acidic soils [60, 72], but as pH is highly similar in both investigated sites (Supplementary Table 1), it does not seem likely that this factor is correlated to the higher number of I.1c sequences detected in Control soils. Lanzén et al. [73] reported higher abundances of I.1c thaumarchaeota in soils covered by denser vegetation in comparison to recently cleared sites, and considering that frequent fires drastically change Cerrado's vegetation structure and composition [33], it is possible that denser wood coverage may lead to higher I.1c subgroup abundance in soils from the site long protected from fires. However, quantitative studies are necessary to better understand the possible roles of fires in different Thaumarchaeota subgroup abundance in Cerrado soils.

Interestingly, *amoA* comparisons showed higher AOA diversity (Table 2) and a greater number of unique OTUs (Figure 3(b)) in soils from the frequently burned site even though analysis of 16S rRNA gene sequences did not reveal such differences (Table 1, Figure 3(a)). Furthermore, although all thaumarchaeal 16S rRNA sequences clustered within I.1b and I.1c subgroups, an *amoA* OTU identified in Control samples was strongly affiliated with *Ca*. Nitrosotalea devanaterra, an organism related to the I.1a lineage. These results highlight the importance of using different molecular markers to better describe environmental microbial communities, an aspect that is being increasingly discussed [12, 74, 75]. The detection of many *amoA* sequences not grouped with any previously cultured AOA, especially in soils from the Control area (Figure 5), suggests the presence of yet unknown AOA in Cerrado soils.

The estimations of greater *amoA* diversity and OTU numbers in soils from the Burned area are consistent with the detection of a higher number of I.1b 16S rRNA thaumarchaeal sequences in the same condition, given that this group is extensively associated to ammonia oxidation in terrestrial habitats [58, 76, 77]. Moreover, lower ammonium concentrations were detected in soils from the biennially burned area, and considering that lower ammonium availability tends to favor AOA growth in soils [78, 79], this factor could have contributed to the higher *amoA* diversity found in this site.

Composition differences in soil AOA and AOB (ammonia-oxidizing bacteria) communities were detected in Australian wet sclerophyll forest areas protected from fires or submitted to biennial burning treatments [80], with shifts in archaeal *amoA* abundance and genotypes, as well as changes in AOB communities, which could result in nitrogen cycling disturbances. However, the differences observed were mainly attributed to alterations detected in soil key parameters between the evaluated sites. Thus, frequent fires in different ecosystems may lead to long-term effects in soil AOA communities through different mechanisms.

There are few studies describing AOA activity and diversity in Cerrado habitats. It has been shown that nitrification rates are generally low in soils from this biome [81] and low AOA and AOB abundances have been reported in soils from a native field of a Cerrado phytophysiognomy denominated *Campo sujo* [82]. However, it is worth pointing out that, despite the identification of short-term modifications of ammonia oxidizer abundance and soil N dynamics in a managed system within the Cerrado biome [82], there are currently no studies exploring the effects of natural ecological events on nitrifier communities in Cerrado soils, especially concerning AOA. Thus, the present study suggests a possible effect of changes in vegetation cover caused by repeated fires on these communities. More studies are necessary to assert the roles of natural ecological events such as seasonality and different fire regimes on archaeal communities in this biome, as well as their ecological roles.

## 5. Conclusion

Our study revealed that I.1b and I.1c Thaumarchaeota are dominant in typical Cerrado *sensu stricto* soils from areas long protected from fire or submitted to frequent fire regime. Although total archaeal community richness and diversity inferred by 16S rRNA genes do not seem to greatly differ between the investigated conditions, *amoA* gene analyses revealed higher diversity and unique OTU numbers in soils from the biennially burned area. Possible differences in relative abundance of thaumarchaeal subgroups I.1b and I.1c were also observed, with higher numbers of I.1c sequences detected in soils long protected from fires and I.1b sequences in the area frequently burned. Our results suggest that alterations in vegetation structure caused by frequent fires could likely cause long-term effects in the composition of archaeal communities in Cerrado soils, especially regarding potential ammonia oxidizers. Further studies are required to determine the extent to which frequent fires could influence archaeal abundance and potential roles in nitrogen cycling in soils from this unique Brazilian biome.

## Figures and Tables

**Table 1:** *α*-Diversity analysis of archaeal 16S rRNA gene sequences from Cerrado *sensu stricto* soils in both conditions analyzed in this study. C = Control area, protected from fire for 20 years, and Q = Burned Area, biennially burned. Measures were calculated independently for each replicate and the mean and standard deviation (SD) of the indicators are shown.

Area	OTU observed	Chao1	ACE	Shannon	Coverage (%)
C	11.0 ± 2.0	18.8 ± 13.3	22.7 ± 15.3	1.958 ± 0.13	91.3 ± 5.0
Q	11.3 ± 2.1	16.0 ± 6.2	15.8 ± 1.6	1.948 ± 0.43	91.3 ± 1.2

**Table 2:** *α*-Diversity analysis of archaeal *amoA* gene sequences from Cerrado *sensu stricto* soils in both conditions analyzed in this study. C = Control area, protected from fire for more than 20 years; Q = Burned Area, biennially burned. Measures were calculated independently for each replicate and the mean and standard deviation (SD) of the indicators are shown.

Area	OTU observed	Chao1	ACE	Shannon	Coverage (%)
C	2.3 ± 0.6	2.3 ± 0.6	2.3 ± 0.6	0.400 ± 0.16	95.3 ± 8.1
Q	5.7 ± 2.1	6.4 ± 3.2	8.0 ± 4.7	1.104 ± 0.32^∗^	93.0 ± 7.0

^∗^Significant differences detected in Welch's *t*-test (*p* < 0.05).

## Data Availability

Sequences obtained in this study were deposited in the GenBank dataset under accession numbers KR828099-KR828564 (16S rRNA gene) and KR828565-KR828812 (*amoA* gene).

## Supplementary Materials

Supplementary Materials Supplementary Table 1: physicochemical parameters of the Cerrado sensu stricto soils from the Control area (C) and the Burned area (Q) at the Reserva Ecológica do IBGE, Brasilia, Brazil. Asterisk indicates statistical differences identified in Welch's *t*-test analysis. Supplementary Table 2: number of high-quality sequences for each 16S rRNA and amoA gene library evaluated in this study. Supplementary Figure 1: rarefaction curves (with 97% sequence similarity) for archaeal 16S rRNA gene sequences from Cerrado sensu stricto soils sampled in the Control (C) and Burned (Q) areas at the Reserva Ecológica do IBGE, Brasilia, Brazil. Supplementary Figure 2: nonmetric multidimensional scaling (NMDS) plot of archaeal communities (inferred by 16S rRNA genes) from soils of Cerrado sensu stricto protected from fires (C) and submitted to biennial fires (Q) at the Reserva Ecológica do IBGE, Brasilia, Brazil.
