# Supplementary material for: Long-Term Effects of Periodical Fires on Archaeal Communities from Brazilian Cerrado Soils
Source: Archaea. 2019 Jan 27;2019:6957210. doi: 10.1155/2019/6957210 (PMC6369511; doi:10.1155/2019/6957210)
Supplement: Supplementary Materials — Supplementary Table 1: physicochemical parameters of the Cerrado sensu stricto soils from the Control area (C) and the Burned area (Q) at the Reserva Ecológica do IBGE, Brasilia, Brazil. Asterisk indicates statistical differences identified in Welch's t-test analysis. Supplementary Table 2: number of high-quality sequences for each 16S rRNA and amoA gene library evaluated in this study. Supplementary Figure 1: rarefaction curves (with 97% sequence similarity) for archaeal 16S rRNA gene sequences from Cerrado sensu stricto soils sampled in the Control (C) and Burned (Q) areas at the Reserva Ecológica do IBGE, Brasilia, Brazil. Supplementary Figure 2: nonmetric multidimensional scaling (NMDS) plot of archaeal communities (inferred by 16S rRNA genes) from soils of Cerrado sensu stricto protected from fires (C) and submitted to biennial fires (Q) at the Reserva Ecológica do IBGE, Brasilia, Brazil. [file 6957210.f1.docx]

**Supplementary Material**

**Long Term Effects of Periodical Fires in Archaeal Communities from Brazilian Cerrado Soils**

**Suplementary Table 1.** Physicochemical parameters of the Cerrado *sensu stricto* soils from the Control area (C) and the Burned area (Q) at the Reserva Ecológica do IBGE, Brasilia, Brazil . Asterisk indicates statistical differences indentified in Welch t-test analysis.

| **Parameter** | **Area** | |
| --- | --- | --- |
|  | C | Q |
| pH (H20) | 4.97 ± 0.05 | 4.93 ± 0.05 |
| P (ppm) | 3.73 ± 0.49 | 4.46 ± 0.40 |
| Ca (cmol_c_/dm^3^) | 0.73 ± 0.15 | 0.60 ± 0.10 |
| Mg (cmol_c_/dm^3^) | 0.2 ± 0.00 | 0.2 ± 0.00 |
| K (cmol_c_/dm^3^) | 0.13 ± 0.00 | 0.13 ± 0.00 |
| Na (cmol_c_/dm^3^) | 0.13 ± 0.05 | 0.10 ± 0.00 |
| Al (cmol_c_/dm^3^) | 0.96 ± 0.11 | 0.75 ± 0.07 |
| H + Al (cmol_c_/dm^3^) | 9.46 ± 0.40 | 8.6 ± 0.69 |
| Sum Bases (cmol_c_/dm^3^) | 1.16 ± 0.28 | 1.00 ± 0.15 |
| CEC (cmol_c_/dm^3^) | 10.66 ± 0.57 | 9.66 ± 0.57 |
| C (g/kg) | 31.13 ± 3.78 | 28.96 ± 2.45 |
| N (g/kg) | 3.06 ± 0.37 | 2.73 ± 0.11 |
| NO_3_ (g/kg) | 1.60 ± 0.14 | 1.36 ± 0.28 |
| NO_2_ (g/kg) | 0.80 ± 0.14 | 0.70 ± 0.10 |
| NH_3_ (g/kg) | **1.30 ± 0.14*** | **0.66 ± 0.15** |
| OM (g/kg) | 53.56 ± 6.52 | 49.8 ± 4.21 |
| Cu (ppm) | 0.54 ± 0.09 | 0.51 ± 0.05 |
| Fe (ppm) | 1084 ± 73.36 | 999 ± 63.63 |
| Mn (ppm) | 13.63 ± 1.34 | 17.48 ± 1.27 |
| Zn (ppm) | 1.23 ± 0.18 | 1.49 ± 0.12 |

**Supplementary Table 2.** Number of high quality sequences for each 16S rRNA and *amoA* gene library evaluated in this study.

| Gene | Clone Library | Number of sequences |
| --- | --- | --- |
| **16S rRNA** | Ca | 80 |
|  | Cb | 79 |
|  | Cc | 74 |
|  | Qa | 82 |
|  | Qb | 101 |
|  | Qc | 50 |
| ***amoA*** | amo_Ca | 44 |
|  | amo_Cb | 45 |
|  | amo_Cc | 39 |
|  | amo_Qa | 47 |
|  | amo_Qb | 46 |
|  | amo_Qc | 30 |


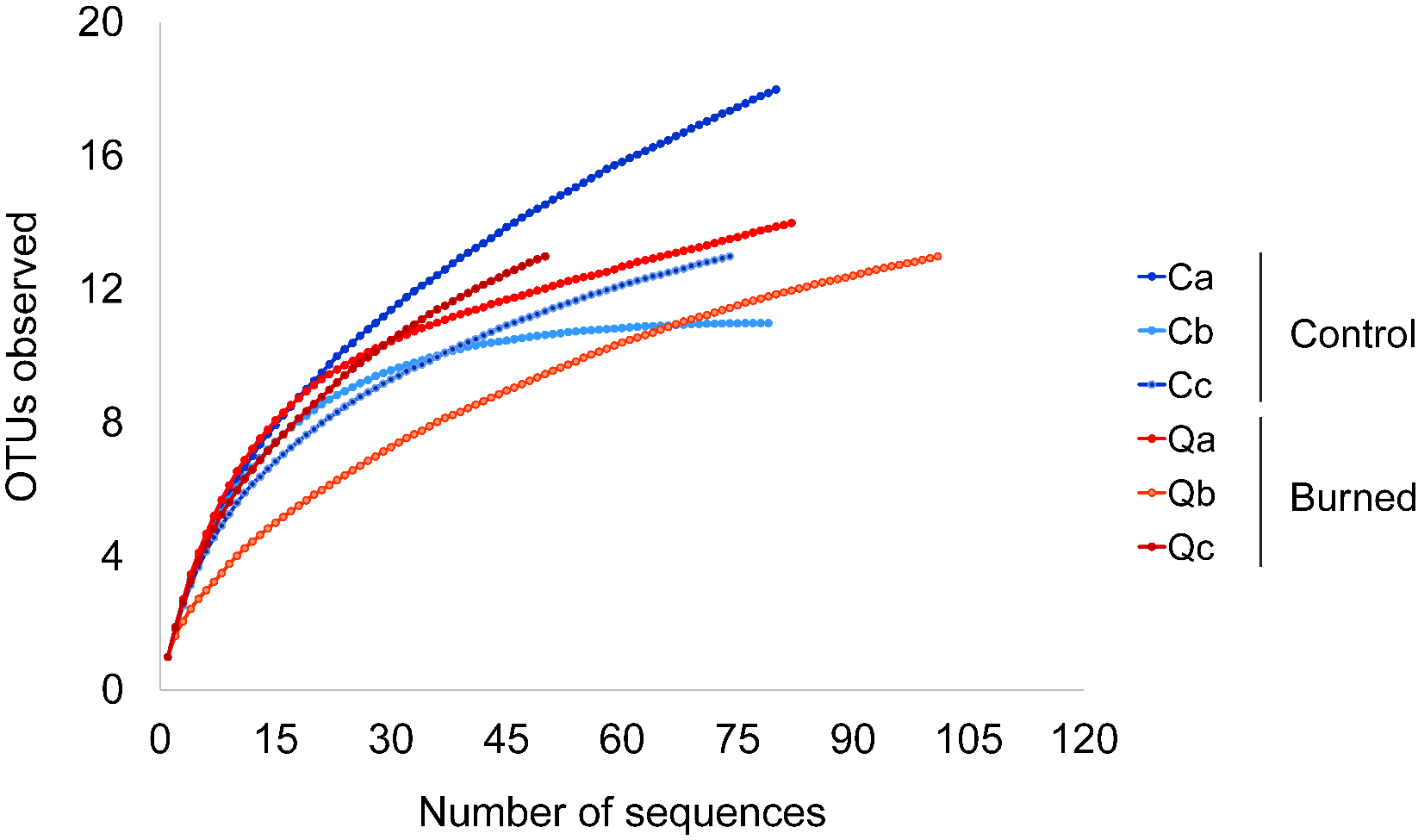


**Supplementary Figure 1**. Rarefaction curves (with 97% sequence similarity) for archaeal 16S rRNA gene sequences from Cerrado *sensu stricto* soils sampled in the Control (C) and Burned (Q) areas at the Reserva Ecológica do IBGE, Brasilia, Brazil.


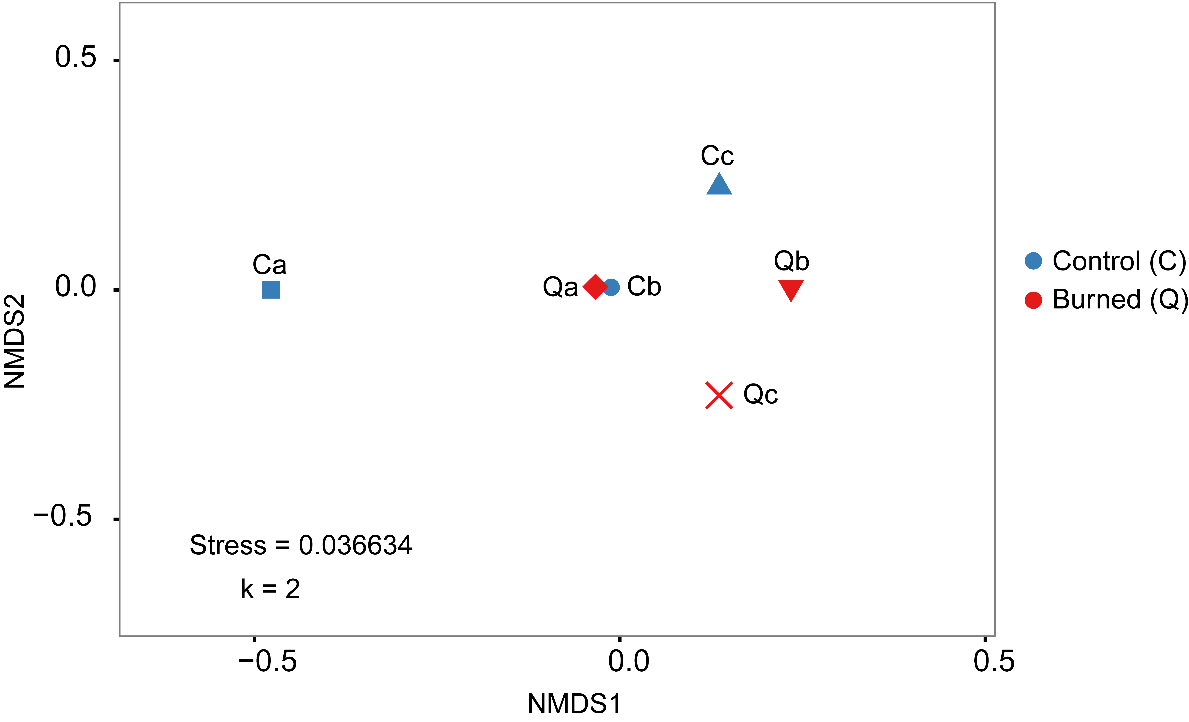


**Supplementary Figure 2**. Non-metric multidimensional scaling (NMDS) plot of archaeal communities (inferred by 16S rRNA genes) from soils of Cerrado *sensu stricto* protected from fires (C) and submitted to biennial fires (Q) at the Reserva Ecológica do IBGE, Brasilia, Brazil.
